# Effectiveness of medicines review with web-based pharmaceutical treatment algorithms in reducing potentially inappropriate prescribing in older people in primary care: a cluster randomized trial (OPTI-SCRIPT study protocol)

**DOI:** 10.1186/1745-6215-14-72

**Published:** 2013-03-13

**Authors:** Barbara Clyne, Marie C Bradley, Susan M Smith, Carmel M Hughes, Nicola Motterlini, Daniel Clear, Ronan McDonnell, David Williams, Tom Fahey

**Affiliations:** 1 Health Research Board (HRB) Centre for Primary Care Research, Royal College of Surgeons in Ireland (RCSI), Beaux Lane House, Lower Mercer Street, Dublin, Ireland; 2 School of Pharmacy, Queen’s University Belfast (QUB), University Road, Belfast Northern BT7 1NN, Ireland; 3 Department of Geriatric and Stroke Medicine, Royal College of Surgeons in Ireland (RCSI), Beaumont Hospital, Beaumont Road, Dublin 9, Ireland

**Keywords:** Multifaceted intervention, Potentially inappropriate prescribing, Primary care, Randomized controlled trial

## Abstract

**Background:**

Potentially inappropriate prescribing in older people is common in primary care and can result in increased morbidity, adverse drug events, hospitalizations and mortality. In Ireland, 36% of those aged 70 years or over received at least one potentially inappropriate medication, with an associated expenditure of over €45 million.

The main objective of this study is to determine the effectiveness and acceptability of a complex, multifaceted intervention in reducing the level of potentially inappropriate prescribing in primary care.

**Methods/design:**

This study is a pragmatic cluster randomized controlled trial, conducted in primary care (OPTI-SCRIPT trial), involving 22 practices (clusters) and 220 patients. Practices will be allocated to intervention or control arms using minimization, with intervention participants receiving a complex multifaceted intervention incorporating academic detailing, medicines review with web-based pharmaceutical treatment algorithms that provide recommended alternative treatment options, and tailored patient information leaflets. Control practices will deliver usual care and receive simple patient-level feedback on potentially inappropriate prescribing. Routinely collected national prescribing data will also be analyzed for nonparticipating practices, acting as a contemporary national control. The primary outcomes are the proportion of participant patients with potentially inappropriate prescribing and the mean number of potentially inappropriate prescriptions per patient. In addition, economic and qualitative evaluations will be conducted.

**Discussion:**

This study will establish the effectiveness of a multifaceted intervention in reducing potentially inappropriate prescribing in older people in Irish primary care that is generalizable to countries with similar prescribing challenges.

**Trial registration:**

Current controlled trials ISRCTN41694007

## Background

### Prescribing in older people

Older people are among the biggest consumers of healthcare services, particularly drug therapy [1]. They tend to have multiple conditions, requiring multiple drug treatments [2]. They also experience age-related changes in physiology and body composition that influence the body’s ability to process medications efficiently, in terms of both pharmacokinetics (the body’s ability to absorb, distribute, metabolize and excrete a drug) and pharmacodynamics (the drug’s physiological effects) [3,4]. Thus, prescribing for older people is a complex and challenging task, with the potential for adverse outcomes including drug-drug interactions, adverse drug reactions and potentially inappropriate prescribing (PIP) [5].

The term ‘potentially inappropriate prescribing’ covers a number of suboptimal prescribing practices, particularly the use of medicines that introduce a greater risk of adverse drug-related events where a safer and equally effective alternative is available to treat the same condition [6]. Inappropriate prescribing in older people can result in increased morbidity, adverse drug events, hospitalizations and mortality [7-9]. Potentially inappropriate prescribing may be measured with explicit (criterion-based) or implicit (judgment-based) tools [9]. A recently developed explicit process measure, the Screening Tool of Older People’s Prescriptions (STOPP), has been published for use in European settings [10].

When these criteria were applied to an Irish pharmacy claims database (containing the prescription records of 97% of those aged ≥70 nationally), it was found that 36% of those aged ≥70 years received at least one potentially inappropriate medication. Total PIP expenditure was estimated at over €45 million (or 9% of expenditure on pharmaceuticals in that age group) [11]. The clinical and economic burden of PIP is an important public health concern and it is important to minimize PIP where possible, to increase patient safety and encourage cost-effective prescribing.

### Interventions to change prescribing

Changing professional practice is a complex and difficult task. While the usefulness of behaviour change theory in intervention and implementation research has been questioned, [12,13] there is general consensus that interventions are more likely to have an impact when they target all stages of behaviour change [14]. Studies to date have yielded mixed results in terms of which intervention types are most effective in improving prescribing and there is no one interventional strategy that has proved to be most effective [15]. Strategies shown to be effective for improving prescribing outcomes include educational outreach visits (academic detailing) [16] and interventions involving a pharmacist [17]. Pharmacist services, such as conducting medicines reviews or providing advice to general practitioners (GPs) may lead to improvements in prescribing outcomes, including more appropriate prescribing in older people [9,17-21]. Patient-mediated interventions do not consistently show effects in improving prescribing [22]; however, evidence suggests that providing patients with information is important. Patient information leaflets may be helpful in improving patient outcomes, and older people appreciate receiving brief, clearly written information leaflets [23,24]. A number of commentators have argued that a multifaceted intervention, an approach that combines a number of techniques within a single intervention [20], may be more likely to work than any one single intervention [9,25,26].

### Study aim and objectives

The aim of this study is to determine the effectiveness and acceptability of a complex, multifaceted intervention in reducing the level of PIP in primary care. The intervention combines academic detailing, medicines review with web-based pharmaceutical treatment algorithms that provide recommended alternative treatment options, and tailored patient information leaflets. The intervention development was informed by the Medical Research Council (MRC) guidelines for the development and evaluation of randomized controlled trials (RCTs) [27,28]. It was piloted with a group of five GPs and found to be feasible and acceptable within this group.

Secondary objectives are to evaluate the effect of the intervention on patient outcomes in terms of the number of GP visits, the number of hospitalizations, patient well-being, beliefs about medicines and health status. The views and experiences of participants concerning the possible reasons for the intervention to be effective or ineffective will be explored using qualitative methodology. In addition, an economic evaluation will be carried out.

## Methods/design

### Trial design

**Figure 1:**
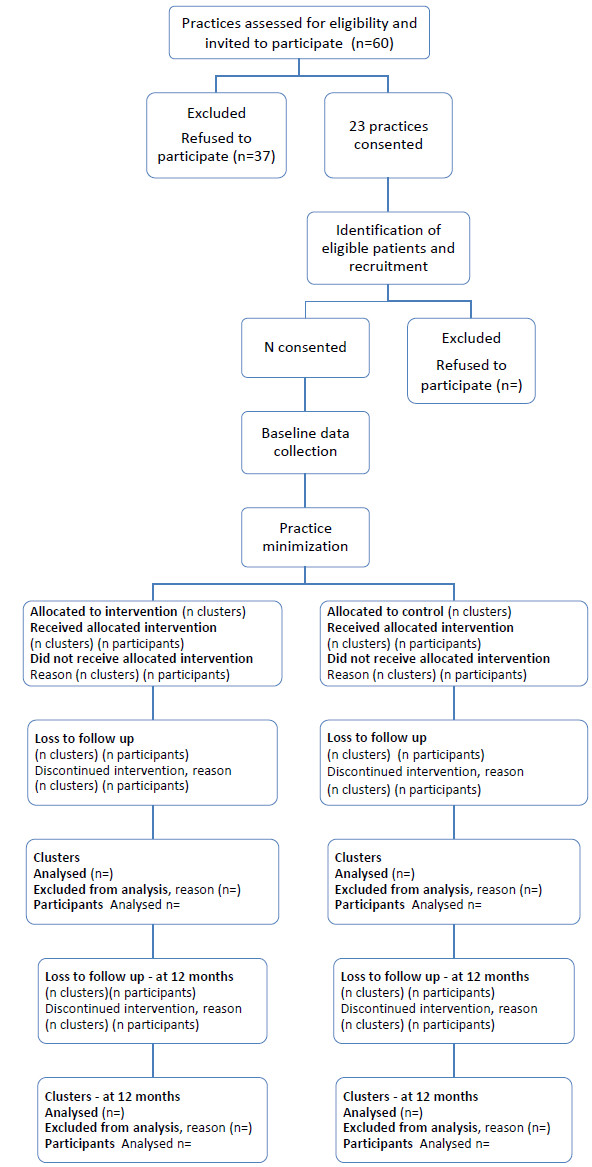
Flow of practices and patients through RCT.

The OPTI-SCRIPT study is a pragmatic two-arm cluster RCT, incorporating qualitative analysis. Qualitative approaches can contribute in several ways to the development and evaluation of health interventions; they have been used during intervention development and piloting and will be used to undertake a process evaluation of the trial [29]. The study design was developed in line with the Consolidated Standards of Reporting Trials (CONSORT) statement extension to cluster RCTs [30]. A cluster design was chosen to avoid the possibility of contamination across arms. A GP who is treating both intervention and control patients might find it difficult to behave differently towards each group [31]. The trial will be conducted in primary care with GP practices as the unit of randomization. A practice can be a single-handed GP or can comprise two or more GPs (group practices); however, not all GPs in group practices may wish to participate. Participation may be decided by one GP on behalf of the group practice, in which case that GP will be the main point of contact for the study. We will keep a record of participating GPs. Participation in the intervention arm will be defined as attendance at the academic detailing visit and undertaking medicines reviews, while participation in the control group as providing patient prescription data. Practices that do not meet these criteria will be considered lost to follow-up (see Figure 1). The intervention will be delivered at the GP level while the unit of analysis will be at the individual patient level, in terms of medicines prescribed, adjusting for clustering.

### Study population

Practices are eligible to participate if they have approximately 80 or more older patients (aged ≥70 years) on their patient panel (based on the need to recruit 10 patients per practice, and allowing for refusals) and they are based in the greater Dublin area (to facilitate the academic detailing process).

Practices are excluded where they have been involved in the development and piloting of the intervention or other concurrent medication quality related studies.

Patients will be considered eligible if they are aged 70 years or over and they are being prescribed one or more selected potentially inappropriate prescriptions on a repeat basis (only patients with an existing PIP will be included, as the intervention is specifically targeting the management of PIP).

Patients will be excluded if they have significant mental or physical illness that is likely to impair their ability to participate in the study, or they are unable to attend the GP surgery for consultation (for example, they are nursing home residents) or they are participating in another medication quality related study.

### Recruitment and allocation

Potentially eligible practices (based in the greater Dublin area) will be identified from the Health Research Board (HRB) Primary Care Research Centre research network. All eligible practices will be invited to participate by an email (or letter where email address is unavailable), which will include a study information leaflet outlining steps of the intervention and availability of continuing medical education (CME) points for participation. When a practice agrees to participate, a member of staff (for example, a GP, practice manager or nurse) will be selected by the practice. The research team will instruct the designated person on how to identify a random sample of 50 patients aged 70 years and over from the patients of participating GPs within the practice. They will pseudo-anonymize the records by assigning the patients a study ID and send a copy of the pseudo-anonymized prescription records to the research team, where a research pharmacist will generate a list of potentially eligible patients, that is, patients with PIP. A maximum of ten patients per practice is required; if more than ten patients are identified, ten patients will be selected at random from the list of eligible patients. Eligible patients will be sent a letter of invitation, a patient information leaflet and a consent form, asking them to participate and answer a questionnaire. Prior to practice allocation, baseline patient data (including prescription data, process-of-care measures and patient-reported outcomes – see sections on outcomes and data collection) will be collected. Practices will then be assigned to intervention or control using minimization. This approach offers the advantage of ensuring balance between the groups [32] in terms of prognostic factors: in this case, practice size (number of whole-time-equivalent GPs) and practice location (urban or rural, where an urban area is defined as a relatively small centre of population, with 5000 or more residents [33]). A chart of the flow of participants through the study is presented in Figure 1. Because of the nature of the intervention, it is not possible to blind GPs or participants to the intervention.

### Intervention

The intervention consists of academic detailing, medicines review with web-based pharmaceutical treatment algorithms and tailored patient information leaflets. The academic detailing will involve a research pharmacist visiting intervention GPs in their own practices. The visit will include a short educational presentation about PIP as a concept, the criteria used to measure it and a summary of studies conducted in Irish primary care on the topic, as knowledge of PIP may be a barrier to appropriate prescribing in older patients [34]. Subsequent to this, practices will be asked to complete ten medicines reviews within a 6 to 8 week period, with a reminder issued if they are not completed within that period. An extended period may be negotiated, should a practice require it. During the medicines review, the GPs will use web-based treatment algorithms specifically designed for this study and accessible using a link and designated password. The algorithm will guide the process from the GP perspective, and does not incorporate patient involvement. It has a page-by-page structure, which will be completed when the GP fills in a review outcome form, detailing decisions made by the GP and patient together, including the reasons for maintaining a PIP, which is a key element of this study. Once the review outcome form has been filled in, the medicines review is complete. The medicines review will take place in the GP practice and will be scheduled at a date and time that is convenient to both the GP and the patient. In group practices where more than one GP is participating, the reviews may be divided between them in a manner that is most suitable to the practice workload. The pilot study indicated that the preparation for the review might be more time consuming for the GP than usual but there was no indication that the consultation itself would be significantly longer. These components are presented in Table 1.

**Table 1:** Intervention components

**Intervention component**	**Description**
Academic detailing	A research pharmacist will visit the intervention practices. During the academic detailing, the pharmacist will:
	• Discuss the concept of PIP with the GPs, focusing on the prevalence and consequences of PIP in primary care
	• Discuss the pharmaceutical treatment algorithm
	• Discuss the medicines review process
	• Demonstrate the web-based platform for accessing the pharmaceutical treatment algorithm for use in a medicines review with each participant patient
Medicines review with web-based pharmaceutical treatment algorithms	GPs will be asked to:
	• Schedule a medicines review for the patient’s next appointment
	• Log on to the designated website using individualized user-names and passwords
	• Access the individualized web-based pharmaceutical treatment algorithms for each patient during the review
	• Conduct a medicines review following the page-by-page web-based pharmaceutical treatment algorithms. Each pharmaceutical treatment algorithm has the following structure:
	Section A: The individual PIP with reason for concern
	Section B: Alternative pharmacological and nonpharmacological treatment options
	Section C: Background information (where relevant)
	• Complete the process by submitting the review outcome form for each PIP per patient
	Each GP will also be provided with a full, paper-based compendium of pharmaceutical treatment algorithms for reference
Patient information leaflets	For every alternative therapy option, a brief patient information leaflet has been written. These leaflets describe the PIP and the reasons why it may be inappropriate. They also outline the alternative therapies the GP may offer instead

Note: The decision on whether to follow the recommended treatment alternatives will be at the discretion of the GP, weighing up the risks and benefits and patient preference.

### Control group: usual care with simple feedback

The control-group GPs will continue to provide usual care but will also be provided with simple feedback. Data for patients in the control group will be reviewed during recruitment and a personalized patient list for the ten recruited patients will be given to the GP. The list will summarize the medication class to which the individual patient’s potentially inappropriate medication belongs, not the specific PIP and will not provide actionable recommendations for change. Participants will not receive an academic detailing visit, will not be prompted to carry out a medicines review with the individual patients, and will not have access to the pharmaceutical treatment algorithms with alternative therapy options. GPs will continue to provide usual care. In terms of repeat prescribing for public general medical services (GMS) patients, this means that a GP can give a prescription on a monthly basis or for a maximum of three months. At present, the Health Service Executive Primary Care Reimbursement Services (HSE-PCRS) has an on-line GP Application Suite where GPs can review administrative information on their GMS patient panel. They can also access prescribing analysis reports, which contain detailed financial and cost information related to their prescribing. Specific prescribing alerts and recommendations for older patients are not provided.

### Contemporaneous national control HSE-PCRS dataset

The control arm will receive simple feedback about their patients based on baseline data collection. Feedback has been found to promote slight improvements in professional practice but is most effective when it is provided intensively [35,36]. By participating in this research, the control group might also alter their behaviour, resulting in changes in prescribing patterns (that is, the possible Hawthorne effect). To address this, we will also analyze national prescription patterns for all GMS doctors via the HSE-PCRS prescription database after the trial. This is a national prescribing database based on GP and pharmacy claims in a number of community schemes, including the GMS scheme. Data from the PCRS GMS database can be used to compare practices participating in the study with nonparticipating practices, acting as a contemporary national control. Some 330,000 people aged 70 and over were eligible for the GMS scheme in 2009 [37]. Comparisons with previous PIP patterns nationally [11] will determine whether there have been changes over time at the population level.

### Outcome measures

The study focuses on a select number of PIP criteria identified in the literature that have been determined to be of clinical relevance by academic GPs and pharmacists, and are prevalent in Irish primary care (see Table 2).

**Table 2:** Selected prescribing criteria and indicators

**Criterion**	**Concern**	Prevalence in Ireland*
PPI for peptic ulcer disease at full therapeutic dosage for >8 weeks	Earlier discontinuation or dose reduction for maintenance or prophylactic treatment of peptic ulcer disease, oesophagitis or GORD is indicated	16.69% [11]
		7.68% [49]
		4.06% [10]
NSAID (>3 months) for relief of mild joint pain in osteoarthritis	Simple analgesics are preferable and usually as effective for pain relief	8.76% [11]
		1.05% [49]
		1.26% [10]
Long-term (>1 month), long-acting benzodiazepines, for example, chlordiazepoxide, flurazepam, nitrazepam and clorazepate, and benzodiazepines with long-acting metabolites, for example, diazepam	Risk of prolonged sedation, confusion, impaired balance, falls	5.22% [11]
		5.19% [49]
		3.00% [49]
		6.01% [10]
		9.09% [10]
Any regular duplicate drug class prescription, for example, two concurrent opiates, NSAIDs, SSRIs, loop diuretics, ACE inhibitors. Excludes duplicate prescribing of drugs that may be required on a p.r.n. basis, for example, inhaled β2 agonists (long and short acting) for asthma or COPD, and opiates for management of breakthrough pain	Optimization of monotherapy within a single drug class should be observed prior to considering a new class of drug	4.78% [11]
		2.18% [49]
		6.01% [10]
TCAs with an opiate or calcium channel blocker	Risk of severe constipation	2.05% [11]
		0.37% [49]
Aspirin at dosage >150 mg/day	Increased bleeding risk, no evidence for increased efficacy	1.69% [11]
		0.3% [49]
		0.14% [10]
Theophylline as monotherapy for COPD or asthma	Risk of adverse effects due to narrow therapeutic index	1.18% [11]
		0.56% [10]
Use of aspirin and warfarin in combination without histamine H_2_ receptor antagonist (except cimetidine because of interaction with warfarin) or PPI	High risk of GI bleeding	1.09% [11]
		0.3% [49]
Doses of short-acting benzodiazepines, doses greater than: lorazepam (Ativan®), 3 mg; oxazepam (Serax®), 60 mg; alprazolam (Xanax®), 2 mg; temazepam (Restoril®), 15 mg; and triazolam (Halcion®), 0.25 mg	Total daily doses should rarely exceed the suggested maximums	0.98% [49]
		1.54% [10]
Prolonged use (>1 week) of first-generation antihistamines, that is, diphenhydramine, chlorpheniramine, cyclizine, promethazine	Risk of sedation and anticholinergic side-effects	0.96% [11]
		0.15% [49]
Warfarin and NSAID together	Risk of GI bleeding	0.75% [11]
		1.68% [10]
Calcium channel blockers with chronic constipation	May exacerbate constipation	0.68% [49]
		0.28% [10]
NSAID with history of peptic ulcer disease or GI bleeding, unless with concurrent histamine H_2_ receptor antagonist, PPI or misoprostol	Risk of peptic ulcer relapse	0.67% [49]
		0.42% [10]
Bladder antimuscarinic drugs with dementia	Risk of increased confusion, agitation	0.46% [11]
		0.84% [10]
TCAs with constipation	May worsen constipation	0.45% [49]
		0.14% [10]
Digoxin at a long-term dosage >125 μg/day (with impaired renal function)	Increased risk of toxicity	0.36% [11]
		0.15% [49]
		0.55% [10]
Thiazide diuretic with a history of gout	May exacerbate gout	0.36% [11]
		0.45% [49]
		0.14% [10]
Glibenclamide (with type 2 diabetes mellitus)	Risk of prolonged hypoglycaemia	0.29% [11]
		0.22% [49]
Aspirin with a past history of peptic ulcer disease, without histamine H2 receptor antagonist or PPI	Risk of bleeding	0.22% [49]
		0.28% [10]
Prochlorperazine (Stemetil®) or metoclopramide with parkinsonism	Risk of exacerbating parkinsonism	0.21% [11]
TCAs with dementia	Risk of worsening cognitive impairment	0.18% [11]
		0.28% [10]
TCAs with glaucoma	Likely to exacerbate glaucoma	0.14% [11]
		0.07% [49]
TCAs with cardiac conductive abnormalities	Pro-arrhythmic effects	0.14% [10]
Long-term corticosteroids (>3 months) as monotherapy for rheumatoid arthritis or osteoarthritis	Risk of major systemic corticosteroid side-effects	0.14% [10]
Bladder antimuscarinic drugs with chronic prostatism	Risk of urinary retention	0.14% [10]
NSAID with heart failure	Risk of exacerbation of heart failure	0.07% [49]
		0.14% [10]
TCAs with prostatism or prior history of urinary retention	Risk of urinary retention	0.07% [49]
		0.14% [10]
Systemic corticosteroids instead of inhaled corticosteroids for maintenance therapy in COPD or asthma	Unnecessary exposure to long-term side-effects of systemic steroids	0.07% [49]
		0.56% [10]
Bladder antimuscarinic drugs with chronic glaucoma	Risk of acute exacerbation of glaucoma	<0.01% [11]
NSAID with SSRI	Increased risk of GI bleeding	N/A
Bladder antimuscarinic drugs with chronic constipation	Risk of exacerbation of constipation	N/A
Prednisolone (or equivalent) > 3 months or longer without bisphosphonate	Increased risk of fracture	N/A
NSAID with ACE-inhibitor	Risk of kidney failure, particularly with the presence of general arteriosclerosis, dehydration or concurrent use of diuretics	N/A
NSAID with diuretic	May reduce the effect of diuretics and worsen existing heart failure	N/A

ACEI, angiotensin-converting-enzyme inhibitor; COPD, chronic obstructive pulmonary disease; GI, gastro-intestinal; GORD, gastro-oesophageal reflux disease; N/A, not available; NSAID, nonsteroidal anti-inflammatory drug; PPI, proton pump inhibitor; p.r.n., *pro re nata*, as needed; SSRI, selective serotonin reuptake inhibitor; TCA, tricyclic anti-depressant. *Prevalence: the proportion of the study population with one or more potentially inappropriate medications.

The primary outcomes to be determined are the proportion of participant patients with PIP (as a composite measure, that is, any number of PIP criteria as listed in Table 2, to address the issue of multiple PIP in individual patients) and the mean number of potentially inappropriate prescriptions per patient.

Secondary outcomes (summarized in Table 3) will include individual measures of the composite measure, that is, drug-specific outcomes, process evaluations, process-of-care measures and patient-reported outcomes (Health status (EQ-5D) [38], Patients’ Beliefs about Medicine Questionnaire (BMQ) [39], Well-being Questionnaire (WBQ-12) [40]).

**Table 3:** Secondary outcome measures

**Secondary outcome**	**Measure**
Drug-specific outcomes	The absolute number of PIPs per patient of the top five occurring PIP drugs: [11]
	Proton pump inhibitor (PPI) for peptic ulcer disease at full therapeutic dosage for >8 weeks
	Long-term (>3 months) use of NSAIDs for relief of mild joint pain in osteoarthritis
	Long-term (>1 month) use of long-acting benzodiazepines, for example, chlordiazepoxide, flurazepam, nitrazepam, chlorazepate and benzodiazepines with long-acting metabolites for example, diazepam
	Any regular duplicate drug class prescription
	TCAs with an opiate or calcium channel blocker
	Mean number of PIPs per patient of the top five PIP drugs (as above)
Patient-reported outcomes	Health status (EQ-5D)
	Patients’ Beliefs about Medicine Questionnaire (BMQ)
	Well-being Questionnaire (WBQ-12)
Process-of-care measures	Number of GP visits (6 months prior to enrolment and at 4 and 12 month follow-up)
	Number of hospital admissions (6 months prior to enrolment and at 4 and 12 month follow-up
Process evaluations	Decisions made per PIP
	Number of times alternatives were prescribed
	Reported primary reason for decision made for example, risks outweigh benefits, patient preference, hospital/consultant initiated

### Data collection

Prescription data, process-of-care measures and patient-reported outcomes will be collected at baseline and on intervention completion, that is, the point at which all ten reviews have been completed (this must be within a 6 to 8 week period). Follow-up data will also be collected 12 months after the intervention completion. Data will be collected from the following sources:

#### GP medical chart

Patient records will be used to collect the drug-specific outcome data for all participants at baseline and follow-up. Process-of-care data, such as health-service utilization (for example, the number of GP visits) will also be collected for intervention and control patients.

#### Questionnaire

Patient-reported outcomes for intervention and control will come from questionnaire data, which will be collected at baseline and follow-up, using a postal questionnaire and telephone follow-up for nonresponders. The questionnaire will be used to collect personal and demographic data, economic data, and health-service utilization data along with data from the EQ-5D, BMQ and WBQ-12.

#### Evaluation data

Process-measure data for the intervention group will be collected by outcome forms completed by GPs at the end of each of the ten medicines reviews they conduct for the ten recruited patients. In single-handed practices, the same GP will conduct the reviews and complete the outcome forms. In group practices, where more than one GP is participating in the study, the reviews may either be conducted by an individual GP nominated by the practice or be shared between the GPs, with GPs completing outcome forms for the patients they reviewed. Semistructured qualitative interviews will also be conducted with both GPs and patients after the intervention, to evaluate the intervention (see below for more detail). GPs in the control arm will also be interviewed, to ascertain any potential impact of the feedback they receive, based on the baseline data.

### Plan of analysis

The minimization process will ensure balance between treatment groups in terms of certain prognostic factors. Descriptive statistics will be used to evaluate differences in other baseline characteristics between participating physicians and patients in the two arms of the trial. The primary analysis will be carried out using multilevel modeling (such as mixed linear effects modeling or generalized estimating equations [41]) to control for the effects of clustering and baseline differences. All analysis will be conducted under the intention-to-treat principle.

Subgroup analyses will be performed for the primary outcome to assess whether the intervention varies by practice size or GP characteristics, such as sex or number of PIP drugs. As this is a pragmatic trial, a secondary, per-protocol analysis will also be conducted. This form of analysis includes only those participants who completed the treatment protocol originally allocated, providing results on the efficacy of the trial [42].

### Sample size

As all the patients in this study will be selected on the basis of already having one or more potentially inappropriate prescriptions, the sample size calculations are based on a 100% prevalence rate. Separate sample size calculations were performed for the two primary outcomes:

#### Proportion of participant patients with PIP

The calculation is based on demonstrating a clinically relevant 10% absolute reduction (from 100% to 90%) in the proportion of PIP with 80% power and a statistical significance of 5% (one-sided), between the randomized groups. With a cluster design, the assumption that individual outcomes are independent of each other does not hold, as participants in the same cluster may respond in the same way. The sample size, therefore, needs to be adjusted to reflect this by use of the intraclass correlation coefficient (ICC) [43]. We used an ICC of 0.025, based on an ongoing unpublished observational study of an elderly cohort in the HRB Centre for Primary Care Research. With a maximum of ten patients per cluster and factoring in a loss to follow-up of 10%, a total of 22 GP practices and 212 patients will be required.

#### Mean number of PIP per patient

An ongoing unpublished observational study of an elderly cohort in the HRB Centre for Primary Care Research estimates a mean number of 1.45 inappropriate prescriptions per patient. To demonstrate a 30% relative reduction in the mean number of PIP (equivalent to a mean of 1.02), with 80% power and a statistical significance of 5% (two-sided), between the randomized groups, with a maximum of ten patients per cluster and factoring in a loss to follow-up of 10%, a total of 14 GP practices and 132 patients will be required.

These calculations indicate that we would need at least 22 practices and 212 patients to detect a difference between the intervention and control arms for both of our primary outcome measures. On the basis of these calculations, we aim to recruit at least 22 practices, with 10 patients per practice, giving a total of 220 patients. With this sample size, we would have at least 80% power to demonstrate a 10% absolute reduction in the proportion of PIP and a 30% relative reduction in the mean number of PIPs. Based on existing evidence that suggests that simple, less intensive feedback does not alter prescribing behaviour [35,36] we have not anticipated an improvement in the control arm. However, we will monitor for this in the parallel process evaluation.

### Data management and protection

A trial steering committee will be established. The aim of the trial is to identify older patients with existing PIP. These patients will be known to the research team by study ID number only. One member of the research team (BC) will have access to patient contact details for follow-up data collection purposes. The GP remains responsible for all treatment decisions made. Informed consent will be sought from all study participants. All data collected will be stored on a secure, password-protected server. All interviews will be audio recorded and transcribed, the digital recording overwritten and the transcripts pseudo-anonymized and stored on a secure, password-protected server.

The academic detailing will demonstrate the process of the medicines review with the intervention practices but the research team will not monitor how the GP implements the study protocol after this, other than to remind the practices to complete the process within the allotted period. This study is pragmatic in nature, measuring the intervention’s effectiveness in real clinical practice.

### Ethical approval

Ethical approval was granted by the Research Ethics Committee of the Irish College of General Practitioners (ICGP). At the request of the Ethics Committee, some changes were made to the study protocol. The patient information letter and questionnaire were rewritten to be clearer and simpler for an older audience. Initially, it was proposed that a member of the research team (BC) would become a research agent of the practice, in order to minimize the effort required by the practice staff to recruit patients [44]. However, the ethics committee requested that the practices should be responsible for the patient consent process. The committee also requested that any prescribing pattern of concern identified by the research team should be referred to an external academic GP with no involvement in the trial to assess the case and to determine the necessary next steps; this arrangement has been put in place.

### Qualitative evaluation

A qualitative process evaluation will be conducted to explore participant attitudes towards the intervention and the experience of the intervention delivery. Specifically, semistructured interviews will be conducted with a sample of participants, both GPs and patients, from the intervention arm. The interviews will be structured using a topic guide, which will be developed with the stages of the interview in mind (introducing the research, beginning the interview, and so on) [45]. These interviews will address such research questions as:

1. What are the views of the participants about the acceptability, effectiveness and sustainability of the intervention?

2. What barriers, if any, were experienced by GPs in relation to implementing the alternative recommended treatments?

3. How did patients respond or react to the idea of altering their medication regimes?

4. Was the medicines review viewed as a useful exercise for the patient or the GP, or both?

5. In what ways might the intervention be modified or adapted to maximize its effectiveness in routine care?

A random sample of participants in the intervention arm will be invited to participate. The number of interviews required to reach data saturation (where no new themes emerge) will be considered, alongside feasibility issues (resources and timing), but a sample of 10 to 15 patients and 10 GPs is proposed [46]. For individual patients, the interviews will take place within one month of the medicines review. For GPs, the interviews will be conducted within one month of them completing their assigned reviews, that is, after all ten reviews for single-handed practices and after completion of assigned reviews in group practices. The interviews will be conducted either in person (in a setting of the participant’s preference) or via telephone. Telephone interviewing is generally used in qualitative research where time or cost is an issue and there is evidence that there is little difference in the answers obtained this way [47]. In this case, scheduling face-to-face interview time with GPs and older patients may be difficult, so telephone interviewing is an option. In addition, as part of the process evaluation, we will conduct brief telephone interviews with the control-group GPs to ascertain any potential impact of the feedback they receive based on the baseline data. Control-group participants will not be required to document any of the decisions made with regard to the control data, so this approach may be more prone to recall bias. However, we consider it a suitable option, given the limited time available in busy GP practices. All interviews will be audio recorded (on loudspeaker for telephone interviews) and transcribed. The data will be collated and a thematic analysis will be conducted. There are four main steps to conducting a thematic analysis:

1. Collect the data.

2. Identify patterns and themes (repetition).

3. Collate related patterns into subthemes.

4. Interpret themes in light of a literature review [48].

NVivo 9 will be used to assist with organizing the data for analysis.

### Economic evaluation

A health economic analysis will be conducted following the RCT to explore the direct costs of the intervention and link these to its potential effectiveness. We will compare the direct costs of delivering the OPTI-SCRIPT intervention as an alternative to usual care. Economic analysis is particularly important in relation to quality of prescribing, owing to the considerable costs invested by healthcare systems in medicines and their prescribing. There are potential cost savings from reducing doses and quantities of inappropriately prescribed medications, and from reducing potential adverse events associated with suboptimal prescribing. There is also potential for cost increases if the appropriate alternative medicine recommended is more expensive. Therefore, it will be important to determine the cost/benefit ratios for any changes made in medicines prescribed as a result of the intervention.

Cost-effectiveness analysis will be undertaken, in which effectiveness will be measured in terms of the reduction in the proportion of potentially inappropriate prescriptions. Direct-cost data will be calculated for all the health resources consumed. All contacts with the health service will be recorded and valued, including GP visits, hospital attendances, hospital admissions and drug prescriptions.

## Discussion

Prescribing for older patients is a complex and challenging task. The literature to date demonstrates that high levels of PIP exist among older people in Ireland [49]. This creates an increased clinical and economic burden with an impact on other patient outcomes, such as increased hospitalizations and mortality. As the proportion of older patients in the population increases and the necessity for pharmaceutical therapy intensifies, it is crucial to find ways to ensure the safety and quality of prescribing in primary care. Currently, no one interventional strategy has proven to be the most effective in addressing PIP. This study is seeking to determine the effectiveness of a complex, multifaceted intervention in reducing the level of PIP in primary care.

The use of the MRC guidelines for the design and evaluation of complex interventions is a strength of this study. The study is innovative in that focuses on a number of PIP criteria that have been determined to be of clinical relevance and high prevalence by academic GPs and pharmacists, rather than applying all criteria from a specific list. Clinically relevant alternatives have also been provided, ensuring that where PIP has been highlighted, actionable recommendations have been made available to the prescriber.

There are some practical limitations to the OPTI-SCRIPT intervention. The identification of PIP in patients could be carried out by a pharmacist who could apply the criteria to patient records but this would require a formalization of the role of the pharmacist within the GP team to enable access to patient records, affecting both cost and service delivery. There are also implications for data protection when a person external to a practice, such as a pharmacist, requires access to patient records. This process could, ideally, be automated, and incorporated into the workflow of the various practice-management software systems used in primary care, along with the treatment algorithms. However, as with all computerized prompts, this would have to be carefully designed to avoid the danger of ‘alert fatigue’, which could become an issue. In addition, a medicines review process is not standard practice in Irish primary care as it is in other countries, such as the UK, where the National Service Framework for older people recommends that all people over the age of 75 should have their medicines reviewed at least once a year [50]. Were such a process to be introduced in Ireland, an agreement would have to be made about reimbursement mechanisms. The majority of people aged 70 and over in Ireland are entitled to free, state-funded GP care and medications (public patients). A small minority of this age group are private patients (approximately 5%), and therefore pay for their own medical care. If a medicines review process were to be introduced into standard care, an agreement would have to be reached as to whether it would be entirely state-funded or whether private patients would have to incur the costs of such a service personally. Should the OPTI-SCRIPT intervention be found to be effective, these issues would need to be taken into consideration prior to its implementation into routine care. In summary, with a growing population of older people, this study will provide evidence concerning the suitability of implementing such an intervention in the Irish Primary Care Sector for older populations.

### Trial status

At the time of submission of this article, 22 GP practices had been recruited. Patient identification and recruitment was just commencing.

## Abbreviations

ACEI: Angiotensin-converting-enzyme inhibitor; BMQ: Beliefs About Medicine Questionnaire; CME: Continuing medical education; CONSORT: Consolidated Standards of Reporting Trials; COPD: Chronic obstructive pulmonary disease; GI: Gastro-intestinal; GMS: General medical services; GORD: Gastro-oesophageal reflux disease; GP: General practitioner; HRB: Health Research Board; HSE-PCRS: Health Service Executive Primary Care Reimbursement Services; ICC: Intraclass correlation coefficient; ICGP: Irish College of General Practitioners; MRC: Medical Research Council; N/A: Not available; NSAID: Nonsteroidal anti-inflammatory drug; PIP: Potentially inappropriate prescribing; PPI: proton pump inhibitor; p.r.n.: *pro re nata*, as needed; QUB: Queen’s University Belfast; RCSI: Royal College of Surgeons in Ireland; RCT: Randomized controlled trial; SSRI: Selective serotonin reuptake inhibitor; STOPP: Screening Tool of Older People’s Prescriptions; TCA: Tricyclic anti-depressant; WBQ-12: Well-being Questionnaire.

## Competing interests

The authors declare that they have no competing interests.

## Authors’ contributions

All authors conceived the development of the intervention and the study design. BC, SS, TF, CH, MB prepared the protocol and contributed to drafting the paper. NM provided statistical advice. RM led the IT design of the study. TF is the principal investigator. The other members of the OPTI-SCRIPT study team are Fiona Boland, Janine Glover and Mary-Claire Kennedy. All authors reviewed and approved the final version of this manuscript.
